# Electroencephalography (EEG) Measures of Neural Connectivity in the Assessment of Brain Responses to Salient Auditory Stimuli in Patients with Disorders of Consciousness

**DOI:** 10.3389/fpsyg.2016.00397

**Published:** 2016-03-22

**Authors:** Victoria Lord, Jolanta Opacka-Juffry

**Affiliations:** Department of Life Sciences, University of RoehamptonLondon, UK

**Keywords:** frontoparietal network, thalamocortical pathways, coherence, neuroplasticity, auditory intervention, music

Disorders of consciousness (DOC) present a clinical challenge in diagnosis, prognosis and defining appropriate treatments that aim at improving the patient's care and quality of life. As there is no universally accepted definition of consciousness, DOC are difficult to define, elucidate and diagnose (Giacino et al., 2014; Schiff et al., 2014).

Modern neuroscience has facilitated a multitude of advanced approaches, models of brain connectivity and techniques, to develop a deeper understanding of the states of consciousness and their transitions, as well as more robust, objective assessment and interventions to be adopted in the clinical environment (Liberati et al., 2014). These could not only enhance and complement diagnosis and assessment but also assist the validation of appropriate rehabilitation interventions, including music therapy.

## The mesocircuit model of thalamocortical connectivity and its application in DOC

The mesocircuit model (Schiff, 2010; Giacino et al., 2014) hypothesizes that the highly dynamic and integrated thalamocortical network is driven by complex and synchronized neuronal firing patterns associated with depolarization of cortical, thalamic and striatal membrane potentials (Giacino et al., 2002, 2014). To illustrate, following a traumatic brain injury (TBI), synchronous activity diminishes across long distance thalamocortical pathways between the prefrontal and parietal cortices, and large-scale dysfunction is seen at the circuit level due to global decreases of excitatory neurotransmission, producing overall changes in cerebral activity levels and reduction in arousal levels (Schiff, 2010; Leòn-Carriòn et al., 2012; Giacino et al., 2014).

This model assumes the key role of the thalamus in integrating thalamocortical pathways, as the thalamus is a central processor not just a simple relay center and performs complex information processing and integration that underlies different mammalian behaviors through corticothalamic feedback input with a high reciprocal connection across cortical areas. The relationship between the thalamus and cortex is bidirectional as the cortex receives thalamocortical inputs and itself projects to the thalamus via corticothalamic fibers (Sherman and Guillery, 2013). This frontocortico-thalamacortical loop responds to the level of synaptic activity; it receives sensory inputs and projects them to the appropriate cortical areas depending on the type of sensory stimulation (e.g., auditory). Such projections are able to regulate cortical states and behaviors: perception and learning and cognition, what we know to be elements of “consciousness” (Tononi and Koch, 2008).

In DOC, structural and functional abnormalities in these pathways reduce the ability to integrate and synchronize cortical areas to process sensory input and couple with executive cognitive functioning (Schiff, 2010; Bagnato et al., 2012; Giacino et al., 2014). Thalamic integrity is related to the severity of DOC in acute and chronic settings (Luckenhoff et al., 2013). DOC states could therefore be interpreted as a “disconnection syndrome” because of impairment in specific cortico-thalamo-coritcal circuits (Monti et al., 2015).

When considering restorative mechanisms in DOC and intra and inter transitioning states, these pathways are implicated in rehabilitation (Laureys et al., 2000; Schiff, 2010; Bagnato et al., 2013). A model of recovery which focuses on connectivity between and within frontal and parietal regions influenced by specific circuit modifications of the thalamocortical pathways is proposed (Laureys and Schiff, 2012; Crone et al., 2014). Evidence for this model comes from studies that show disrupted functional connectivity in a widespread frontoparietal network in patients with impaired consciousness (Luckenhoff et al., 2013; Schiff et al., 2014; Monti et al., 2015) and studies which emphasize the important role of the thalamus (Laureys and Schiff, 2012). Across the network, thalamocortical plasticity may occur through different mechanisms, such as sensory stimulation or deprivation: auditory, visual, cognitive, or somatosensory, impacting thalamocortical arborisation and dendritic spine density (e.g., Bagnato et al., 2013). This has considerations regarding the impact of rehabilitation interventions which could induce neuroplasticity and therefore the functional network of the brain required for synchronized connectivity across the thalamo–cortical network to identify changes and characteristics associated with transitional states of a DOC.

The mesorcicuit model and concept of thalamocortical connectivity in DOC has led to research models which aim to measure brain dynamics in response to different stimulation and at rest states. The aim of this is not only to elucidate the condition but also to develop standardized and robust objective assessment methods that can be adopted in the clinical environment (Liberati et al., 2014).

## Applications of electroencephalography (EEG) to studies of brain connectivity in DOC

Modern neuroimaging and recording techniques such as MRI, fMRI, PET, and electroencephalography (EEG) have facilitated the integration of structural and functional methods enabling a greater understanding of the variation in different states and transitions of a DOC, as well as some individual differentiators within the spectrum (Laureys et al., 2004; Liberati et al., 2014). EEG serves as a direct measure of neuronal activity, which is independent of overt motor communication responses; this is particularly pertinent to studies on DOC where these very functions are severely impaired (Laureys et al., 2004). Thanks to these characteristics, EEG has become a global standardized technique widely used in both clinical and research settings with well-defined criteria for interpretation.

EEG can measure brain signals of neuronal activity over bandwidths of frequency ranges recorded with dozens or hundreds of scalp locations over different timescales; it has particular relevance when considering brain dynamics and the mesocircuit model in DOC. Brain dynamics are measured through signal oscillations which act as communication connections integrating brain functions. They are important for the mediation and distribution of higher functional and synchronized processes in the human brain (Sauseng et al., 2005). Moreover, it has been proposed that brain oscillatory systems act as possible communication networks with relationship to integrative brain functions (Fingelkurts et al., 2013). The advent of EEG digital technology has led to quantitative computational models which analyse dynamic internetwork connectivity within DOC in response to different stimuli—auditory, visual, cognitive, motor, and resting state (Lehembre et al., 2012; Malinowska et al., 2013).

## EEG coherence in DOC

The most accepted measure of interaction and connectivity is that of coherence, a generalization of correlation to the frequency domain. Coherence is used to study the relationship and interactions between EEG channels and between brain regions (Nolte et al., 2004). Coherent neuronal oscillations are seen to correlate with cognitive and behavioral functions, mediating long, and local range communication, impacting synaptic plasticity (Maris et al., 2007; Plankar et al., 2013). EEG coherence is computed between pairs of electrodes, providing information on the extent of synchronization between two time series. A high coherence between signals at different sites of data collection indicates an increased functional connectivity between the regions of interest within the neuronal network, thus identifying a circuit of neural activity; such measures have a temporal definition as they can be collected at various time points (Cohen, 2014). Apart from the obvious advantages of such information, there are also limitations as scalp-recorded EEG measures of coherence do not provide insights into the discrete anatomical and physiological factors that underpin circuit activity (Chorlian et al., 2009).

In DOC, coherence is used to study connectivity and synchronization between brain regions. Correlated activities amongst brain areas or electrodes can determine patterns of functional connectivity, which is known to be impaired in DOC (Noirhomme and Laureys, 2014). Although a wide range of EEG patterns are present in brain injured patients, there are broad regularities that are identifiable across the power spectrum for coma, DOC states and normal wakefulness (Malinowska et al., 2013).

One of the main limitations of this approach is that coherence values must be interpreted carefully, as they can be contaminated by artifacts between electrodes caused by reference and by volume conduction which is pertinent to the DOC population. Volume conduction can impact interelectrode connectivity results due to the spatial autocorrelation between electrodes which measures connectivity between the two brain regions where the electrodes are placed, this is further impacted by skull injury and craniotomy following a TBI (Cohen, 2014). This has been addressed by a modified measurement of coherence that takes into account only the imaginary part of coherence (iCoh; Nolte et al., 2004). Although some neural information may be lost due to removing the real part of coherency, the benefit is that the imaginary part explains only true brain interactions (Lehembre et al., 2012; Cohen, 2014).

Research using the modified imaginary coherence across difference frequency ranges and brain regions in DOC using resting state and diverse stimuli has expanded significantly over the last few years (e.g., Leòn-Carriòn et al., 2012, 2013; Höller et al., 2013; Chennu et al., 2014; Bagnato et al., 2015).

EEG studies of coherence can be used to assess the effects of auditory stimuli in DOCs. One of the most reliably tested methods is using salient auditory stimuli either through music therapy or music intervention that is meaningful or personal to the patient. DOC studies have identified that auditory stimulus with personal and/or emotional meaning produces meaningful results compared to unrelated auditory stimuli (e.g., O'Kelly et al., 2013; Magee et al., 2014; Okumura et al., 2014; Heine et al., 2015).

## EEG coherence following salient auditory stimuli in DOC—current and future directions

**Figure 1:**
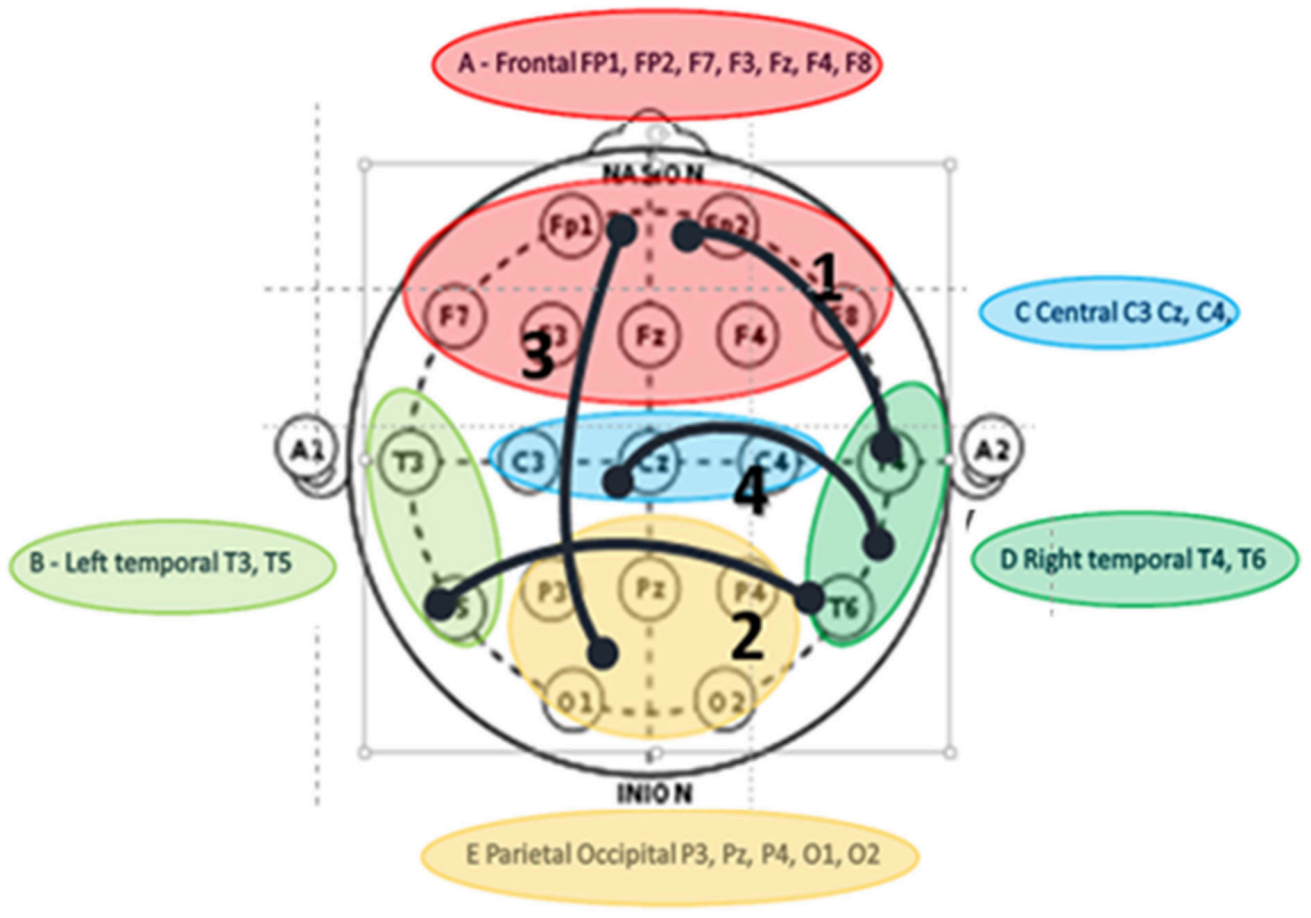
**Interregional Theta connectivity using i-coherence values to represent connections following music therapy intervention in DOC**. Values of coherence not shown as diagram for illustrative purposes only. Paired electrodes groups into 5 anatomical brain regions: “1” indicates strongest regional coherence from right temporal to frontal electrodes; “2” is second coherence from right to left temporal regions; “3” is the 3rd strongest from frontal to parietal/occipital regions and “4” is the fourth level of i-coh between right temporal and central regions. There is no indication of connectivity direction, only interconnectivity (Lord, 2015).

Music therapy is an established rehabilitation intervention in DOC (Magee et al., 2014; Raglio et al., 2014; Magee and O'Kelly, 2015). While there is a large body of literature regarding the brain responses to music (e.g., review by Koelsch, 2014) and music rehabilitation in DOC, as recently reviewed by Kotchoubey et al. (2015), there is a paucity of reports on EEG coherence and music therapy in DOC. A first of this kind of clinical research (O'Kelly et al., 2013) has evaluated the effects of salient auditory stimuli in patients with minimally conscious and vegetative states; which demonstrated theta increases in the frontal and temporal discriminatory activity in response to salient music stimuli. There is an obvious need for longitudinal DOC clinical studies, which analyse synchronized connectivity in response to salient auditory stimuli by means of EEG coherence. To address this, and following on the work by O'Kelly et al. (2013), a current clinical study evaluates the effects of music therapy on regional coherence in patients with DOC in a clinical rehabilitation setting during a course of music therapy applied over 6–18 months (Figure 1) (Lord, 2015). This collaborative research uses the mesocircuit model and thalamocortical synchronization to explore whether increased synchronization in brain connectivity across thalamocortical pathways can be considered as an indicator of increased awareness or consciousness, evaluating the responses to salient auditory stimuli over time.

More research is needed on the mesocircuit model and thalamocortical synchronization to develop an EEG regional coherence methodology in order to analyse and compare cortical regional connectivity in response to rehabilitation interventions, including music therapy. The application of these models in a clinical setting could help develop an EEG regional coherence methodology to analyse and compare cortical regional connectivity in response to music rehabilitation interventions.

It is important to understand if an increase in connectivity across the mesocircuit pathways could be an indicator of the conscious state and neuroplasticity, and whether such a change could monitor the functional recovery in line with the earlier works (Schiff, 2010; Leòn-Carriòn et al., 2012; Bagnato et al., 2013; Chennu et al., 2014; Crone et al., 2014). This approach could also facilitate the assessment of thalamocortical neuroplasticity as a possible effect of music intervention, by analysing regional coherence levels as indicators of awareness and arousal in response to salient auditory stimuli.

## Conclusions

The application of the mesocircuit and thalamocortical connectivty models using EEG coherence measures could lead to a more objective determination of the state of consciousness in patients with DOC and any transitions that may happen across the continuum within the different states through the trajectory of rehabilitation with the contribution of music therapy. It is hoped that the development and adoption of such clinically applicable and complementary tools across DOC patient populations will enhance the existing practizes to provide more translational and differential evaluation of post-injury diagnosis and throughout rehabilitation.

## Author contributions

VL is the lead author. Both VL and JO contributed to the conception and design of this work. VL drafted the manuscript and JO revised it critically for intellectual content. Both authors approved the final version of manuscript. Both VL and JO agree to be accountable for all aspects of the work.

### Conflict of interest statement

The authors declare that the research was conducted in the absence of any commercial or financial relationships that could be construed as a potential conflict of interest.
